# Lung immune incompetency after mild peritoneal sepsis and its partial restoration by type 1 interferon: a mouse model study

**DOI:** 10.1186/s40635-024-00707-7

**Published:** 2024-12-20

**Authors:** Qiuming Meng, Fumiko Seto, Tokie Totsu, Tomoyuki Miyashita, Songfei Wu, Masahiko Bougaki, Michiko Ushio, Takahiro Hiruma, Bruce C. Trapnell, Kanji Uchida

**Affiliations:** 1 Department of Anesthesiology, Graduate School of Medicine, The University of Tokyo, 7-3-1 Hongo, Bunkyo-Ku, Tokyo, 113-0033 Japan; 2 Department of Emergency and Intensive Care Medicine, Department of Emergency and Critical Care Medicine, Fukushima Medical University, Southern Tohoku Research Institute for Neuroscience, Southern Tohoku General Hospital, Fukushima, Japan; 3 Division of Pulmonary Biology, Perinatal Institute, Cincinnati Children’s Hospital Medical Center, Cincinnati, OH USA

**Keywords:** Sepsis, Acute lung injury, ARDS, Innate immunity, Immune suppression, Immune reprogramming, Interferon-β

## Abstract

**Background:**

Sepsis is commonly associated with acute respiratory distress syndrome (ARDS). Although the exaggerated inflammation may damage intact lung tissues, a percentage of patients with ARDS are reportedly immunocompromised, with worse outcomes. Herein, using a murine sepsis model, time-course immune reprogramming after sepsis was evaluated to explore whether the host is immunocompromised. Leukocyte kinetics in the lung tissue were evaluated in a male C57/BL6 mouse model of mild peritoneal sepsis induced by cecal ligation and puncture, with the survival rate exceeds 90%. Lung immune reactivity was evaluated by intratracheal instillation of lipopolysaccharide (LPS; 30 µg). Furthermore, the effect of interferon (IFN)-β in vivo and ex vivo was evaluated.

**Results:**

Four days after sepsis, the lung water content remained high, even among mice in clinical recovery. While monocytes and neutrophils gradually accumulated in the lung interstitium, the inflammatory cytokine/chemokine expression levels in the lungs continued to decline. Intratracheal LPS instillation induced more leukocyte trafficking and protein leakage into the alveoli in the septic lung, indicating more severe lung injury. However, LPS stimulation-associated mRNA expression of *tnf*, *il6*, *ccl2*, and *cxcl1* was suppressed. Intra-alveolar expression of tumor necrosis factor (TNF)-α, interleukin (IL)-6, monocyte chemoattractant protein (MCP)-1, and keratinocyte-derived cytokine (KC) was also suppressed. Monocytes isolated from the lung tissue showed an impaired response in *il6*, *ccl2*, and *cxcl1* to LPS. Systemic IFN-β restored the above impaired regulator function of monocytes, as did coculturing these cells from lung tissue with IFN-β.

**Conclusions:**

Histologically accelerated inflammation and paradoxically suppressed immunological regulator signaling were observed in the early recovery phase of sepsis. This observation may provide a model for the immunologically irresponsive state that occurs in some patients with sepsis. Systemic IFN-β partly restored the post-septic immunocompromised state, indicating its therapeutic potential for the immunosuppressive state seen in some patients with sepsis/ARDS.

**Supplementary Information:**

The online version contains supplementary material available at 10.1186/s40635-024-00707-7.

## Background

Sepsis has a prevalence of 750,000 cases per year in the United States [1] with a mortality rate of 25–50% [2, 3]. Acute respiratory distress syndrome (ARDS) is common [4, 5] and accounts for 32% of all sepsis-related deaths [6]. Despite intense investigation, the primary mechanisms underlying ARDS development are incompletely understood [6], and sepsis-related ARDS mortality has not improved over the past 20 years [7]. While mechanical ventilation improves survival in ARDS patients [8–11], no specific pharmacotherapy has been proved to be effective in clinical use [12–14] and clinical outcomes are generally poor. Early concepts of ARDS pathogenesis held that an exuberant and excessive inflammatory response to the intact lung existed; however, numerous trials using anti-inflammatory treatments have failed [15]. More recently, the ARDS pathophysiology has been considered to involve immunosuppression, which can contribute to increased infection and higher mortality rates [16].

Cecal ligation and puncture (CLP) is a popular murine model of sepsis clinically resembling colon perforation in humans [17]. We previously described a mouse model of sequential sepsis from CLP followed by lung infection that had a high mortality rate due to incomplete neutrophil recruitment as a possible pathogenic mechanism [18]. Systemic interferon (IFN)-β treatment partially restored impaired macrophage function and improved survival [18]. However, there is no clear answer to the question of how changes in the distribution and function of immune cells after sepsis lead to this phenomenon.

In this study, we examined the temporal distribution and function of immune effector cells and lung immune status following the induction of mild peritoneal sepsis by CLP. Lung immune status was evaluated with a biological stimulus (i.e., lipopolysaccharide; LPS) to precisely evaluate the lung immune response. Our hypothesis is that the innate immune remodeling that occurs after sepsis is immunosuppressive. The effects of systemic/topical IFN-β treatment were also evaluated. The results revealed conflicting phenomena of histologic hyperinflammation and an impaired response to biological stimuli (i.e., immune paralysis) in the early recovery phase from sepsis, as well as the therapeutic potential of IFN-β.

## Methods

Additional details are included in the online supplement. The experimental protocols were approved by our institutional review board (#Med-P20-106), conformed to the NIH Guide for the Care and Use of Laboratory Animals (1985 revision), and were conducted in compliance with ethical guidelines issued by the University of Tokyo Graduate School of Medicine.

### Peritoneal sepsis

Peritoneal sepsis was induced in male C57/BL6 mice (age, 8–10 weeks) (CLEA Japan, Tokyo, Japan) by CLP [18]. Briefly, under general anesthesia (2–4% isoflurane), the exteriorized cecum was ligated at 1 cm from the distal pole and punctured with a 23-gauge needle. This procedure is recognized as mild peritoneal sepsis with least severity [19]. The control group only underwent laparotomy and cecum exposure. Murine sepsis scores were monitored every 12 h with the table including seven parameters consisting of spontaneous activity, response to touch and auditory stimuli, posture, respiration rate and quality (labored breathing or gasping), and appearance (i.e., degree of piloerection) [20]. The total score is calculated by assigning a score of 0–4 to each category (i.e., minimum of 0 and a maximum of 28). On the day of sampling, mice were euthanized by exsanguination under deep anesthesia.

### LPS instillation-induced lung injury

On day 4 post-surgery, selected mouse cohorts under general anesthesia with 2% isoflurane received intratracheal (i.t.) instillation of sterile PBS or 30 µg of *Escherichia coli* LPS (O111: B4; Sigma–Aldrich, St. Louis, MO, USA) solution in PBS. One day later, excised lung tissues were fixed, paraffin embedded, sectioned, stained with hematoxylin and eosin, and evaluated under light microscopy (Keyence, Tokyo, Japan).

### Interferon-β administration

A certain proportion of animals were treated with systemic recombinant murine IFN-β (6 µg/kg, R&D Systems, Minneapolis, MN, USA) after CLP. Isolated monocytes from septic lungs were incubated in RPMI media (Life Technologies Corp. Grand Island, NY, USA) with IFN-β (0.125 µg/ml) for 3 h before LPS stimulation.

### Bronchoalveolar lavage fluid (BALF) collection

BALF was collected by three washes with 0.5 ml PBS containing 1 mM EDTA (total 1.5 ml). Cells and supernatants were separated by centrifugation (300 × *g*) for further evaluation.

### Lung immune cell isolation

The right inferior lobe of the lung was enzymatically dissociated with a Mouse Lung Dissociation Kit using a gentleMACS^®^ Octo Dissociator (Miltenyi Biotec, Auburn, CA, USA), following the manufacturer’s instructions. Monocytes were isolated for in vitro assays using an EasySep™ immunomagnetic cell isolation kit (STEMCELL, Vancouver, BC, Canada).

### Flow cytometry

Fluorochrome-conjugated antibodies against the following proteins were used for differential cell counting [21] (Suppl. Figure E3) by a CytoFLEX flow cytometer (Beckman Coulter, Brea, CA, USA): CD45, CD11b, CD11c, SiglecF, Ly6G, Ly6C, CD115, MHCII, CD3, and CCR2 (Suppl. Table E1).

### Cytokine analyses

Cytokines (interleukin [IL]-6, IL-10, tumor necrosis factor [TNF]-α, keratinocyte-derived cytokine [KC], and monocyte chemoattractant protein 1 [MCP-1]) in serum, BALF, and culture media were evaluated by a flow-based cytometric bead ELISA kit (BD Biosciences, Franklin Lakes, NJ, USA), following the manufacturer’s instructions.

### mRNA expression assay

The expression levels of *ccl2*, *cxcl1*, *il6*, *tnf*, and *il10* in lung tissue and monocytes were quantified by TaqMan^™^ Gene Expression assays (Applied Biosystems, Framingham, MA, USA) (Suppl. Table E2) using the StepOne^™^ Real-Time PCR System (Applied Biosystems). The results are expressed as fold changes relative to unstimulated controls for each condition.

### RNA-sequencing

Total RNA was extracted from the left lung tissues from both control mice and mice 4 days after CLP using the phenol–chloroform method, and RNA quality was confirmed (RNA integrity number > 8). mRNA was enriched and used to prepare strand-specific libraries with the NEBNext^®^ Ultra^™^ II Directional RNA Library Prep Kit (New England Biolabs, Ipswich, MA, USA). The mRNA was reverse-transcribed into first-strand cDNA. The cDNA libraries were sequenced on an Illumina NovaSeq 6000 platform for paired-end reads. Raw data were processed using FastQC for quality control, Trimmomatic for trimming, and HISAT2 for alignment to the reference genome. Gene expression levels were quantified with featureCounts. Full details are provided in the supplemental digital content.

### Statistical analysis

To compare survival rates, the log-rank test was conducted. For numerical data, the Shapiro–Wilk test and Levene’s median test were used to assess normality and equal variance, respectively. If the normality and equal variance tests were satisfied, parametric tests were applied (i.e., unpaired *t* test for two-group comparisons and one-way ANOVA followed by the post-hoc Tukey–Kramer honestly significant difference test for multiple group comparisons). Data are presented as the means + SD. If the normality test failed, nonparametric tests were applied (Mann–Whitney *U* test for two-group comparisons and the Kruskal–Wallis *H* test followed by Dunn’s signed rank-sum test for multiple group analyses). Data are presented as the medians (25th, 75th percentiles). All statistical analyses were performed using JMP Pro17.0 (SAS Institute Inc.), and *P* < 0.05 were considered statistically significant.

## Results

### Mouse recovery 4 days after CLP-induced mild peritoneal sepsis

The mild peritoneal sepsis model applied in this study had a survival rate of over 90% (Suppl. Figure E1A). Mice were confirmed to survive beyond postoperative day 4 in pilot experiments. Mice had resumed drinking and eating by postoperative days 1–2, with weight loss ceasing by day 3 (Suppl. Figure E1B), which usually indicates the start of clinical recovery (i.e., return of bowel movement) in patients who have undergone surgery, trauma or seriously ill [22, 23]. Murine sepsis scores [19], which assess severity through activity, response, posture, respiration, and appearance, dropped to zero by day 4, indicating unimpaired activity (Suppl. Figure E1C). These observations suggested that the acute phase of peritoneal sepsis was resolved.

The serum cytokine profile indicated that MCP-1 and KC peaked at day 2, and TNF-α peaked at day 3 (Suppl. Figure E2), then declined, returning to baseline at day 5. Serum IL-6 remained significantly elevated on day 5.

### Post-CLP water content and cellular distribution in the lungs over time

**Fig. 1:**
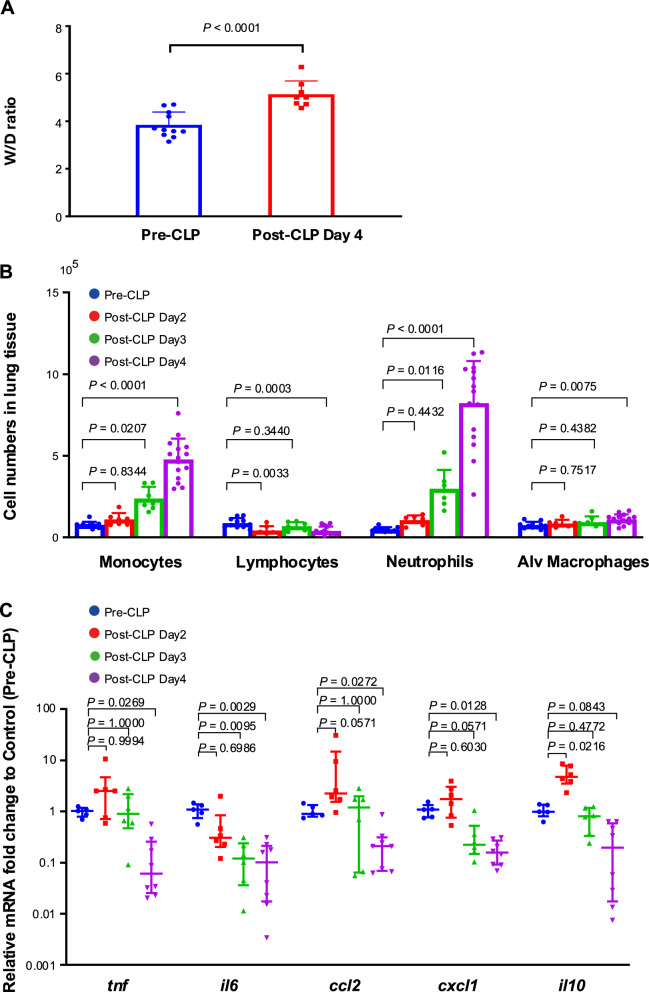
Sepsis-related lung immune reprogramming. **A** Lung wet/dry weight ratios showing a significant increase from pre-cecal ligation and puncture (CLP) to day 4 post-CLP in the CLP group, indicating that the post-CLP lung remained edematous despite clinical recovery. **B** White blood cell populations over time after sepsis. White blood cells in the lung tissue were isolated by enzymatic digestion, counted with a hemocytometer, and differentiated by flow cytometry with immune staining (Figure E3 in the data supplement). **C** Time-course changes in mRNA expression after sepsis. All evaluated cytokines/chemokines except for *il10* exhibited decreased expression over time and significant reductions on day 4 compared with expression pre-CLP. Data are expressed as the mean + SD, accompanied by individual data points (**A**, **B**). Data are expressed as the median ± [25th, 75th percentile], accompanied by individual data points (**C**). Exact *P* values are shown for each comparison

Despite the clinical recovery trend on day 4 post-CLP, the lung wet/dry (W/D) weight ratio was significantly higher than that in controls, confirming the persistent lung edema on day 4 (Fig. 1A).

The sequential changes in the cellular composition of the lung tissue post-CLP (Suppl. Figure E3) revealed a gradual decrease in lymphocytes and a slight increase in alveolar macrophages by day 4, along with significant increases in monocytes and neutrophils by day 4 (Fig. 1B). Despite the interstitial neutrophil and monocyte accumulation, no infiltration in the alveoli was observed, and 95% of the cells recovered from BALF were alveolar macrophages (Suppl. Figure E4).

### Changes in inflammatory mediator expression in the lungs over time

CLP resulted in significant reductions in *tnf*, *il6*, *ccl2*, and *cxcl1* mRNA expression levels in lung tissue over time, with each dropping to one-tenth of their respective pre-CLP level on day 4 post-CLP (Fig. 1C). RNA sequencing results revealed that, in addition to inflammatory cytokines, the expression levels of *p21*, *socs1*, *il4*, *il11*, and *tgf* were significantly decreased on day 4 post-CLP compared with the control. By contrast, only *socs3* showed a significant increase (Suppl. Figure E7).

These results indicated that 4 days after CLP, despite the apparent clinical recovery, the lungs remained edematous, with increased neutrophil and monocyte accumulation in lung tissues. By contrast, the mRNA expression levels of mediators in the lung were downregulated over time.

### Lung damage after LPS instillation

**Fig. 2:**
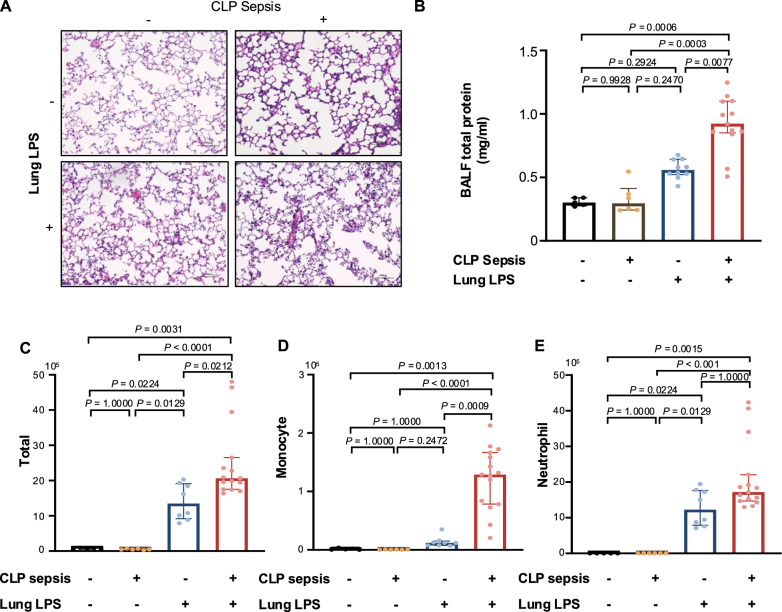
Sepsis aggravates lung injury histologically and biochemically. **A** Representative hematoxylin and eosin-stained images of the lung at 4 day post-cecal ligation and puncture (CLP) with or without intratracheal (i.t.) lipopolysaccharide (LPS) instillation. Compared with the nonintervention control (upper left panel), the 4-day CLP-treated lung showed mild thickening of the interstitium and capillary congestion (upper right panel). At 1 day post i.t. LPS challenge, the lung without CLP showed mild infiltration of inflammatory cells into the alveoli and mild accumulation of edema fluid (bottom left panel). Lungs after 4 days of CLP and 1 day after i.t. LPS exhibited increased migration of inflammatory cells into the alveoli and protein-rich edema fluid in the alveoli observed macroscopically (bottom right panel). **B** i.t. LPS-induced lung fluid protein concentration in the bronchoalveolar lavage fluid (BALF) from mice with sepsis was significantly higher than that in the noCLP group. **C**–**E** I.t. LPS-induced intra-alveolar influx of white blood cells. **C** Sepsis alone did not increase the total number of recovered cells above that of the nonintervention group, whereas LPS administration induced a significant increase in the number of cells migrating into the alveoli. Prior sepsis further significantly (*P* = 0.0212) increased the number of recovered cells. **D**, **E** Sepsis induced a significant increase in migrated monocytes (**D**), whereas neutrophils were not significantly increased (**E**). Data are expressed as the median ± [25th, 75th percentile], accompanied by individual data points. Exact *P* values are shown for each comparison

To examine immune function after the induction of mild peritoneal sepsis, lung damage after LPS challenge was evaluated. Histological examination of the lungs 1 day after LPS stimulation showed alveolar leakage of edematous fluid and inflammatory cell migration into the alveoli in both the CLP and control (noCLP) groups (Fig. 2A). Furthermore, in the LPS-treated CLP group, the amount of protein leakage into the BALF and the number of leukocytes migrating into the alveoli were significantly higher than those in the other groups (Fig. 2B, C). The numbers of neutrophils, the most abundant cell recovered after i.t. LPS stimulation, were not significantly different between the CLP and noCLP groups (Fig. 2E), but the monocyte numbers were significantly higher in the CLP group (Fig. 2D). Based on the amount of protein leakage and leukocyte influx into the alveoli, lung inflammation in response to i.t. LPS stimulation appeared to be more severe in the CLP group than in the other groups.

### Ly6C^lo^ monocyte accumulation in the lungs

**Fig. 3:**
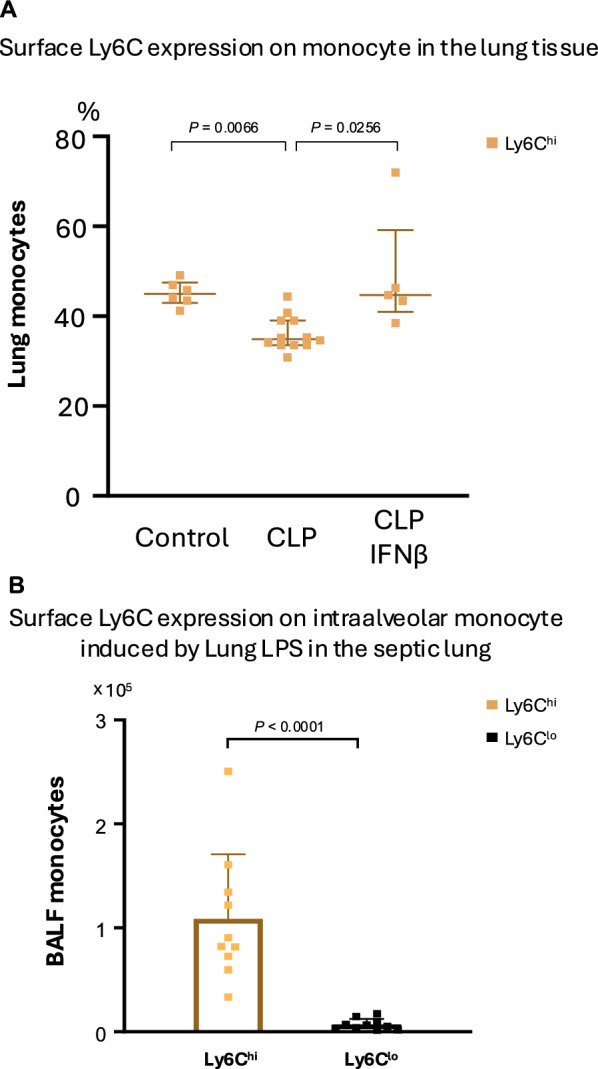
Changes in the monocyte populations in lung tissue after sepsis. **A** Proportions of Ly6C^lo^ and Ly6C^hi^ monocytes in the control group were nearly equivalent (47% vs 53%), whereas these proportions changed to Ly6C^lo^ dominant (67% vs 33%) at 4 days post-cecal ligation and puncture (CLP). The monocyte population returned to pre-CLP levels (50:50 for Ly6C^hi^ and Ly6C^lo^) with systemic interferon (IFN)-β administration. **B** Following intratracheal (i.t.) instillation of lipopolysaccharide (LPS), 95% of monocytes migrating into the alveolar space (BALF) were Ly6C^hi^. Data are expressed as the median ± [25th, 75th percentile], accompanied by individual data points (**A**). Data are expressed as the mean + SD, accompanied by individual data points (**B**). Exact *P* values are shown for each comparison

Monocytes in the lung were further characterized by their expression of the inflammatory monocyte marker Ly6C. Without CLP intervention, monocytes in the lung interstitium consisted of Ly6C^hi^ and Ly6C^lo^, each comprising 50% of the population. Four days after CLP, the population became Ly6C^lo^ dominant (40% Ly6C^hi^ and 60% Ly6C^lo^) (Fig. 3A, data on the far left and in the middle). By contrast, almost all monocytes migrating into the alveoli after i.t. LPS treatment were Ly6C^hi^ (Fig. 3B). Ly6C^hi^ monocytes in the lung were also CCR2^+^ (Suppl. Figure E5).

### Suppressed response to LPS stimulation

One day after i.t. LPS instillation, TNF-α, IL-6, KC, and MCP-1 serum levels were significantly elevated in the control group (noCLP), whereas they were almost absent in the CLP group (Suppl. Figure E6).

**Fig. 4:**
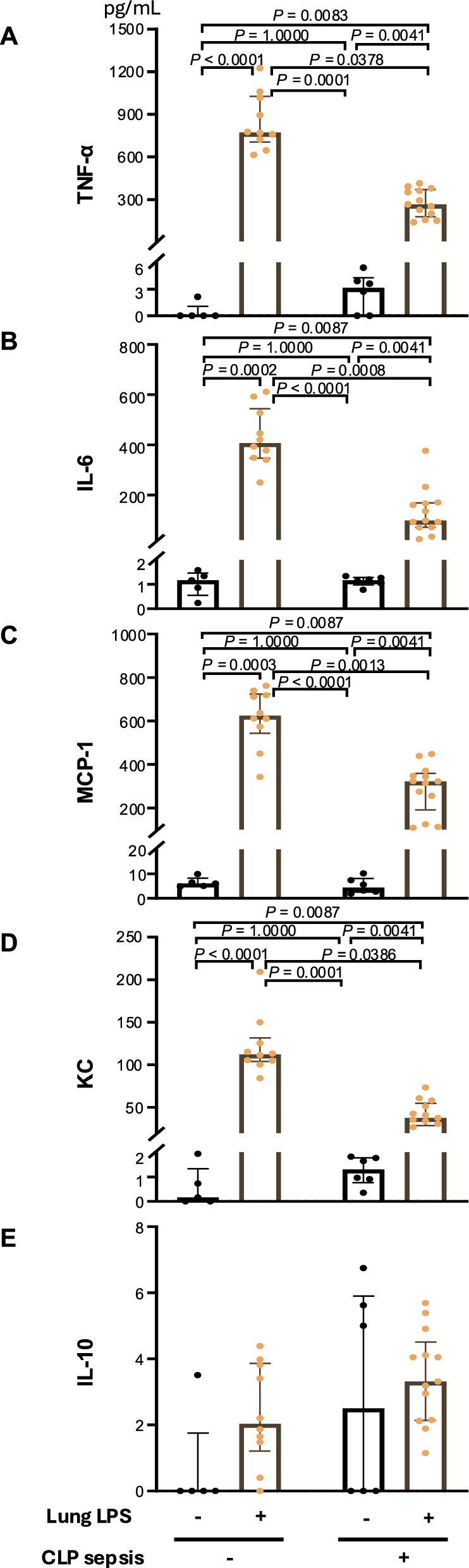
Effect of sepsis on the intra-alveolar cytokine response to intratracheal (i.t.) lipopolysaccharide (LPS) instillation. **A**–**E** I.t. LPS instillation (30 µg in PBS) induced significant increases in the indicated cytokines and chemokines except for IL-10 in both the cecal ligation and puncture (CLP) and noCLP groups compared with their respective controls (i.e., noLPS). However, the magnitude of the increases was lower in the CLP group than in the noCLP group. Data are expressed as the median ± [25th, 75th percentile], accompanied by individual data points. Exact *P* values are shown for each comparison

**Fig. 5:**
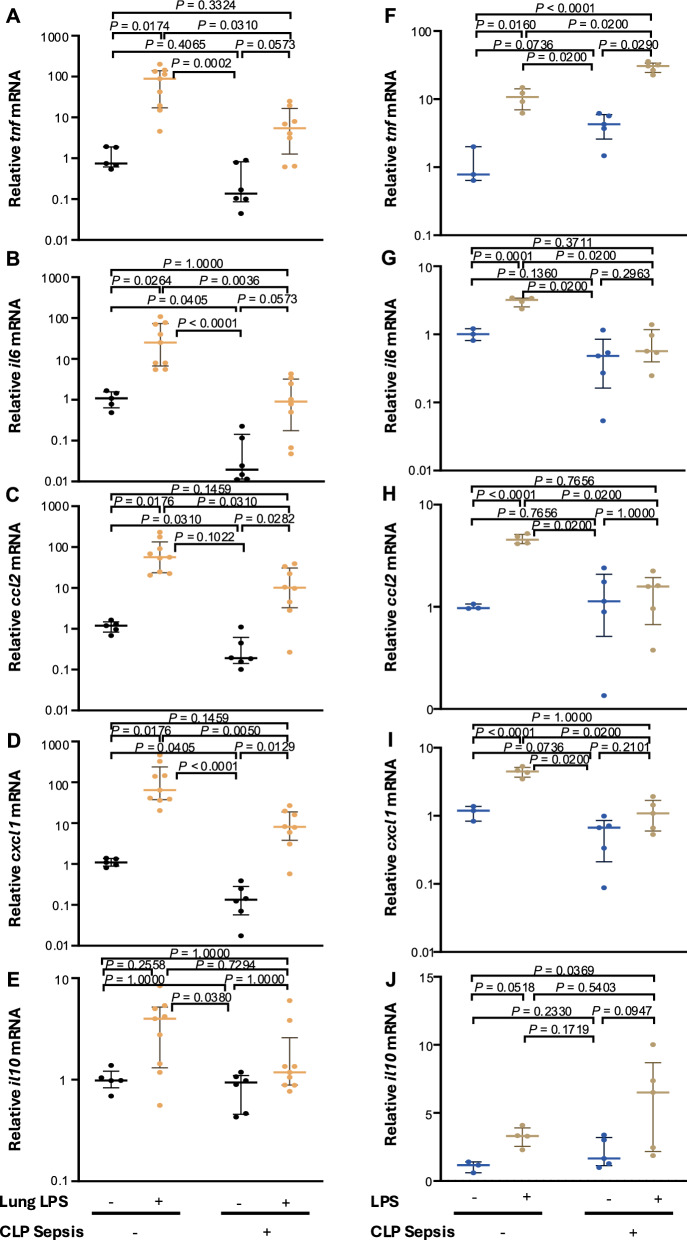
Effect of sepsis on lung cytokine mRNA expression in response to intratracheal (i.t.) lipopolysaccharide (LPS). **A**–**E** mRNA expression of the indicated cytokines and chemokines in the lung tissue of mice with or without cecal ligation and puncture (CLP)-induced sepsis and at 1 day post i.t. LPS instillation (30 µg). In the noCLP group, all five cytokines and chemokines evaluated showed significant increases after LPS stimulation compared with levels in the noCLP without LPS group. Although all cytokines/chemokines except for IL-10 were also significantly increased with i.t. LPS in the CLP group compared with the group without LPS, the magnitude of the increases was significantly lower compared with those in the noCLP + i.t. LPS group. **F**–**J** Real-time PCR evaluation of the mRNA expression of the indicated cytokines and chemokines in monocytes isolated from lung tissues of mice with or without CLP-induced sepsis and stimulated with LPS (10 ng/mL) or PBS in vitro. Similar to the response patterns in lung tissue, the in vitro LPS-induced expression *of il6*, *ccl2*, and *cxcl1* were lower in monocytes isolated from the lungs of the CLP group compared with those from the noCLP group, with the exception of *tnf*, which had higher levels than those in the noCLP group. Data are expressed as the median ± [25th, 75th percentile], accompanied by individual data points. Exact *P* values are shown for each comparison

Intratracheal LPS-induced inflammatory cytokine/chemokine expression in the BALF (i.e., TNF-α, IL-6, MCP-1, and KC) was significantly suppressed in the CLP group compared with the noCLP group (Fig. 4). Furthermore, *il6*, *ccl2*, *tnf*, and *cxcl1* mRNA expression in lung tissue was significantly upregulated by i.t. LPS administration in both the CLP and noCLP groups compared with their respective controls (i.e., noLPS); however, the expression levels of these factors were significantly lower in the CLP group, with each dropping to one-tenth of the respective noCLP level (Fig. 5A–E).

The increases in *il6*, *ccl2*, and *cxcl1* mRNA expression in monocytes treated with LPS in vitro were suppressed in the septic lung (Fig. 5G–J) with the exception of *tnf* expression (Fig. 5F).

### Effect of IFN-β administration on lung immune function

**Fig. 6:**
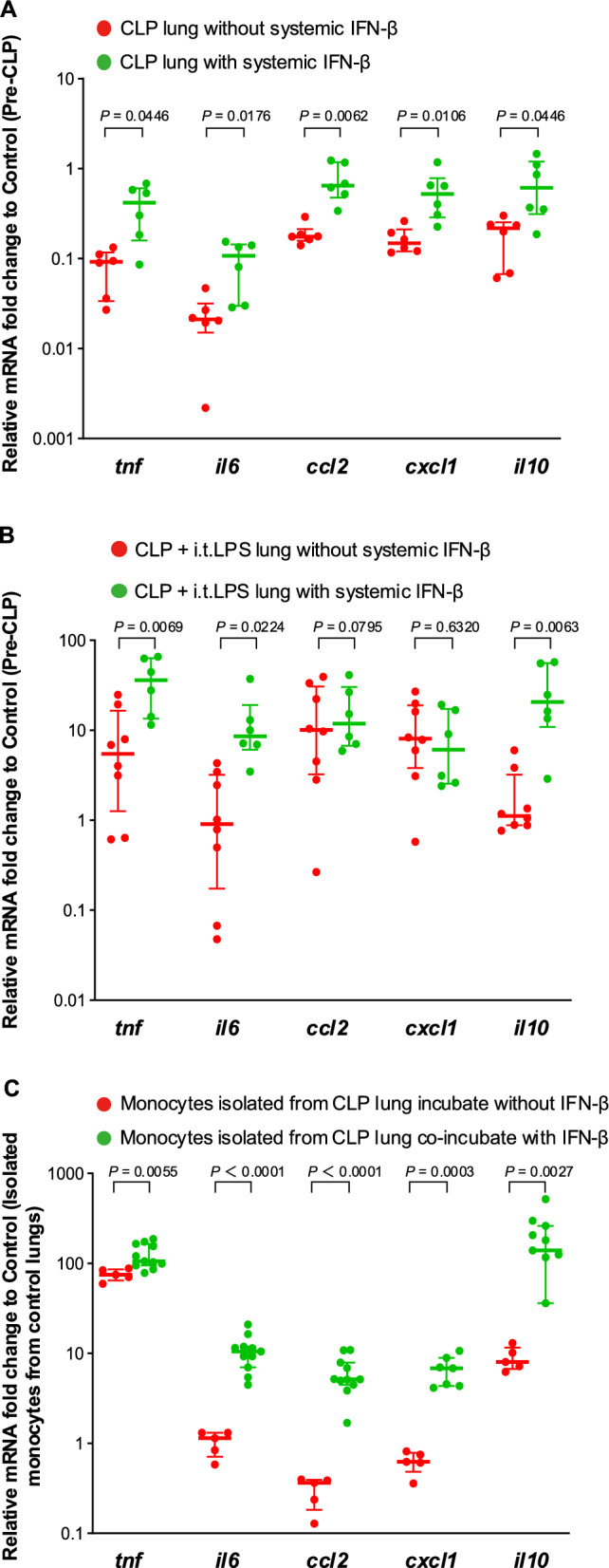
Effect of interferon (IFN)-β administration on lung immune function. **A** mRNA expression levels in lung tissue 4 days after cecal ligation and puncture (CLP) with systemic IFN-β administration 1 h after CLP were significantly higher than those in the CLP without IFN-β group. **B** Expression levels of *tnf* and *il6* in lung tissue, with systemic IFN-β administration 1 h after CLP and 4 days later upon stimulation with i.t. LPS, were significantly higher than those in the CLP without IFN-β group. **C** LPS-stimulated expression of *il6*, *ccl2*, and *cxcl1* in isolated monocytes from the CLP group was restored by coculture with IFN-β. Data are expressed as the median ± [25th, 75th percentile], accompanied by individual data points. Exact *P* values are shown for each comparison

mRNA expression in lung tissue 4 days after CLP with systemic IFN-β was significantly higher than that of the CLP without IFN-β group (Fig. 6A). The expression levels of *tnf* and *il6* in lung tissue after CLP followed by i.t. LPS were significantly higher in the group receiving IFN-β administration compared with the group without IFN-β (Fig. 6B). Monocyte populations in the lung returned to the pre-CLP level (50:50 for Ly6C^hi^ and Ly6C^lo^) (Fig. 3A data on the far right). The LPS-stimulated expression of *il6*, *ccl2*, and *cxcl1* in monocytes isolated from the septic lung was restored by coculture with IFN-β (Fig. 6C).

## Discussion

In this study, we observed immune remodeling in the lung over time in a mouse model of mild peritoneal sepsis. Four days after mild peritoneal sepsis, when mice were in clinical recovery, a significantly elevated lung water content and significant monocyte and neutrophil accumulation in the lung interstitium were observed. Intratracheal LPS-induced lung inflammation appeared to be more severe in the septic lung, as indicated by increased leukocyte trafficking and protein leakage into the alveoli. However, the lung mRNA levels of inflammatory cytokines and chemokines, namely, *tnf*, *il6*, *ccl2*, and *cxcl1*, decreased over time by post-CLP day 4 to approximately one-tenth of their respective pre-CLP levels. The lungs on day 4 post-CLP also showed suppressed *tnf*, *il6*, *ccl2*, and *cxcl1* mRNA expression upon LPS instillation. RNA sequencing results revealed that the expression levels of *p21*, *socs1*, *il4*, *il11*, and *tgf* were significantly decreased on day 4 post-CLP compared with the control, indicating a global suppression of mediators, except for *socs3*. Although the LPS-induced monocyte influx into the alveolar space was increased in the septic lung, the levels of MCP-1, KC, IL-6, and TNF-α expression in BALF were suppressed.

Monocytes isolated from septic lungs and stimulated with LPS in vitro showed increased *tnf* and *il10* mRNA expression, while expression of *il6*, *ccl2*, and *cxcl1* was highly suppressed.

The monocyte proportion in the lung interstitium was shifted to Ly6C^lo^ dominant post-sepsis. Systemic recombinant IFN-β administration to septic mice partially restored lung mRNA expression, reverted the Ly6C^hi^/Ly6C^lo^ ratio to pre-CLP values, and revived LPS reactivity in monocytes isolated from the lungs.

Lung injury as a form of CLP-induced remote organ damage has been reported in many studies [24–27]. In the present study, we characterized this phenomenon through careful evaluation of immunocompetent cell clustering in the lungs over time. Sepsis-mediated disruption of epithelial/endothelial cell integrity has been well documented [26] and was confirmed by the high degree of intra-alveolar protein leakage following LPS stimulation in our study.

We found that the LPS stimulation-induced myeloid cell influx into the alveoli was significantly higher in the CLP group than the control group, despite the relatively low KC and MCP-1 levels. This phenomenon implies a disruption in the integrity of the vascular endothelium and the alveolar epithelium during sepsis. By contrast, the cytokine/chemokine profile after LPS stimulation indicated suppression of these factors (Suppl. Figure E8).

In our previous report using the same mild peritoneal sepsis model, we suggested that decreased bacterial clearance due to impaired neutrophil migration caused by decreased KC secretion from the alveoli led to ARDS [18]. Consistent with this, this current study’s findings suggested that the lung injury state, as revealed by inflammatory cell infiltration and protein leakage into the alveoli, may not correspond to a typical hyperinflammatory state, which is usually recognized as increased host-defense capacity including inflammatory cytokine secretion.

In our previous study, i.t. administration of *Pseudomonas aeruginosa* in the same mild peritoneal sepsis model as used in the present study resulted in a mortality rate exceeding 90% [18], whereas in the present study, i.t. LPS instillation, an aseptic irritant, rarely caused death in mice. Therefore, these model mice appeared to have a significant impairment in the host-defense capacity against pathogens, rather than excessive inflammation.

Discrepancies between pathological findings and the immunological response have been demonstrated in recent studies. Analysis of COVID-19 pneumonia patients revealed that the immune phenotype is immunosuppressive, even in fulminant ARDS patients [28]. A randomized controlled trial of simvastatin in ARDS patients has shown that there is an immunosuppressive population and that the drug has little effect in that group [29]. Using mouse models of sepsis with relatively low mortality, previous studies have demonstrated impaired innate immunity up to 4 days after CLP [30–32]. By contrast, other studies have reported evidence of inflammatory activation during this period [25, 33, 34]. We have compiled a summary of studies that evaluated pulmonary immune function in mouse models, including our previous research (Table 1) [18, 25, 30–38]. The findings are inconsistent, with some studies suggesting immune suppression even in the chronic phase (10–21 day post-CLP) [38], while others indicate immune activation [35]. Our current study, which focuses on the later stage of the subacute phase (day 4 post-CLP), demonstrated a decline in immune responsiveness.

**Table 1:** Past publications of animal models evaluating post-sepsis immune function

	Time of evaluation	Sepsis mortality	Parameters evaluated	Parameters for Lung injury/immunity
Reddy et al. 2001 [30]	24 h after CLP	20–30%	Alveolar macrophage cytokines production response to LPS stimulation; Phagocytosis of Alveolar macrophage	Impaired host immune responses to LPS challenge and phagocytic activity
Deng et al. 2006 [32]	24 h after CLP	< 10%	Alveolar macrophage cytokines production response to LPS stimulation; Pulmonary expression of TLR4 signaling pathway	Impaired host immune responses to subsequent LPS or *Pseudomonas aeruginosa* challenge
Muenzer et al. 2010 [31]	1, 4, 7 days after CLP	< 10%	Changes in immune cell numbers; Survival following *Pseudomonas aeruginosa* challenge at 4- and 7-day post-CLP	
Bhargava et al. 2013 [33]	4, 24 h after CLP	< 10%	Kidney and lung injury parameters	Increased MPO activity and percent neutrophil accumulation
Drechsler et al. 2015 [36]	6,24, 48, 72, 96 h after CLP	30–50%	Liver injury parameters; Organ dysfunction score	No development of significant injury
Seemann et al. 2017 [34]	24, 48, 72 h after CLP	< 10%	Serum cytokines; Liver/kidney injury parameters; Clinical severity score	Increased levels of inflammatory cytokines and oxidative stress
Park et al., 2018 [25]	72 h after CLP	100%	Real-time pulmonary endothelial surface layer imaging	Endothelial surface layer degradation, prolonged neutrophil entrapment
Kusakabe et al. 2018 [37]	24 h after CLP	Mild sepsis; < 20% Severe sepsis; 20–40%	Peritoneal/serum cytokines; Peritoneal/serum innate immunity	N/A
Hiruma et al. 2018 [18]	4 days after CLP	< 10%	Peritoneal/serum/BALF cytokines; Phagocytosis of Alveolar macrophage	Impaired KC secretion and bacterial clearance against subsequent *Pseudomonas aeruginosa* challenge
Baudesson de Chanville et al. 2020 [38]	10 days after CLP	< 20%	Ly6^hi^ monocyte and polymorphonuclear redistribution in lung, bone marrow, spleen, blood, liver and kidney	Intravascular Ly6^hi^ monocyte showed limited activation, reduced phagocytosis
Denstaedt et al. 2021 [35]	21 days after CLP	< 20%	Lung leukocyte population and response to LPS stimulation	Accumulated Ly6^hi^ monocyte enhanced lung injury after secondary LPS stimulation
The present study	4 days after CLP	< 10%	Lung monocyte population and response to LPS stimulation	Impaired expression of inflammatory mediators

*CLP* cecal ligation and puncture, *N/A* not available

Series of publications have reported the properties of Ly6C^hi^ and Ly6C^lo^ monocytes; specifically, monocytes recruited by signals such as CCL-2 differentiate into Ly6C^hi^ macrophages, which produce factors such as transforming growth factor-β, platelet-derived growth factor, TNF-α, and IL-1β that activate inflammation [39]. Ly6C^hi^ monocytes have been reported to exacerbate renal injury in sepsis [40], experimental cerebral malaria [41], and lung injury caused by diffuse pulmonary hemorrhage [42]. In the LPS-induced acute lung injury mouse model, Ly6C^+^ monocytes reportedly exacerbated inflammation [43]. In addition, M1-dominant monocytes in the bloodstream have been associated with a worse prognosis in a baboon model of peritoneal sepsis [44]. Denstaedt et al. found Ly6C^hi^ monocyte accumulation in the lungs 3 weeks after CLP, suggesting it as a potential cause of enhanced inflammation after the second insult with LPS stimulation [35]. By contrast, Ly6C^lo^ monocytes exert anti-inflammatory effects, suppress T-cell function through CD52–HMGB1 binding, and express matrix metalloproteinases to prevent fibrosis [39].

Li et al. proposed the idea that Ly6C^hi^ macrophages are of the M1 phenotype and that Ly6C^lo^ macrophages are of the M2 phenotype [39]. Based on previous reports [45], we can reasonably infer that Ly6C^hi^ monocytes are inflammatory, while Ly6C^lo^ monocytes are anti-inflammatory.

Using i.t. LPS, we found that monocytes entering the alveoli were CCR2^+^Ly6C^hi^, indicating the M1 phenotype. Despite this, the TNF-α, IL-6, KC, and MCP-1 levels in BALF were reduced.

It has been reported that monocytes, like macrophages, can exhibit phenotypes other than M1 and M2 [46, 47] and that Ly6C^hi^ and Ly6C^lo^ switching occurs in response to the surrounding environment [48, 49]. Furthermore, the lung environment may change in response to the systemically secreted cytokine environment, with plasticity in monocyte reactivity. These may be the reason for the reduced cytokine secretion by intraalveolar monocytes in the sepsis lung despite being Ly6C^hi^.

In the present study, systemic IFN-β administration restored reduced cytokine mRNA (including *tnf*, *il6*, *ccl2*, *cxcl1*, and *il10*) expression on day 4 post-CLP. The impaired response to LPS in monocytes isolated from septic lungs was restored by coincubation with IFN-β. The Ly6C^hi/lo^ ratio also returned to control levels with systemic IFN-β. We have previously shown that IFN-β promotes re-expression of inflammatory mediators via IFN-α/β receptor in macrophages during the LPS-tolerant phase, when TLR4 signaling is suppressed by SOCS signaling [18, 37]. The observation that, at day 4 post-CLP, the expression of mediators in the lung tissue—both pro-inflammatory and anti-inflammatory—was globally suppressed, with the exception of *socs3*, which was upregulated, supports this interpretation. The present results also suggest that IFN-β partially restores the immunological remodeling of monocytes in septic mouse lungs.

In contrast to the highly suppressed synthesis of *il6*, *ccl2*, and *cxcl1* following CLP, the synthesis of *tnf*, another inflammatory cytokine, was relatively preserved in the present study. We previously reported that alveolar macrophages stimulated with LPS synthesize intracellular *socs3* mRNA, which renders cells tolerant to LPS [37]. SOCS3 suppresses the MyD88-related signaling pathway but not the TRIF pathway among the TLR4 signaling pathways. Since *tnf* mRNA can also be synthesized via the TRIF pathway, preserved TRIF pathway signaling may be the mechanism explaining the observed phenomenon [50, 51].

In the present study, systemic administration of IFN-β restored the suppressed cytokine response without causing excessive activation. Combined with its previously reported ability to inhibit increased pulmonary permeability via CD73 [52], the administration of IFN-β in the current model may enhance host defense mechanisms without inducing further lung injury due to excessive inflammation.

### Clinical implications

Although some patients may certainly benefit from immunosuppressive therapy in severe sepsis-related conditions, the host-defense capacity may be compromised in others; thus, it is crucial to evaluate the immune status of individual patients when implementing immunomodulatory therapy.

Immunological reprogramming after sepsis potentially may not provide an effective biological defense against pathogens in the lung and may represent one mechanism for developing ventilator-associated pneumonia, which frequently occurs in patients recovering from sepsis. To prevent nosocomial pneumonia, restoration of adequate host-defense capacity within the lungs is necessary. Furthermore, the partial restoration of the LPS response capacity in immunocompetent cells by systemic IFN-β administration is a promising result for future clinical applications.

A randomized controlled trial involving ARDS patients classified as moderate to severe according to the Berlin definition failed to demonstrate the efficacy of IFN-β administration [53], leading to recommendations against its routine use until new evidence is reported [54]. In addition, our group's research in a different sepsis mouse model has shown that the timing of administration significantly influences outcomes [37], highlighting the clear need for indicators to identify responsive patient populations.

### Limitations

The sequential sepsis → pneumonia model included only male mice; however, sex is a known risk factor for ARDS [55], and the influence of female hormones has not been examined in this model. A variety of other factors should be considered in a heterogeneous clinical setting.

In the present study, the characterization of Ly6C^hi/lo^ monocytes, including their host-defense capacity and intracellular protein expression, was not performed. This remains a crucial area for investigation in future studies. Furthermore, the tissue concentrations of systemically administered IFN-β, as well as soluble CD73—a marker of IFN-β efficacy [52]—were not measured in this study. The effects of IFN-β on the ARDS mouse model include the CD73-mediated prevention of vascular permeability, as reported by Kiss et al. [52]. Given that our previous study demonstrated attenuated lung injury in a *P. aeruginosa* pneumonia-induced ARDS model, it is anticipated that IFN-β may also exert beneficial effects on lung injury in the current model. This mechanism was not explored in this study but warrants further investigation in future studies.

Minimally invasive surrogate markers to detect the immune status of the host should be investigated for clinical application. The indication for IFN-β administration should be further studied because the reverse effect of immunosuppression by IFN-β, as we have reported in the past [18, 37], may produce opposite results depending on the timing of administration.

## Conclusions

In mice recovering from mild peritoneal sepsis, despite monocyte and neutrophil accumulation in the lungs, the immune response remained suppressed. In vivo administration of IFN-β partially restored the sepsis-induced impaired immune response. These findings suggest that pathological evaluation alone may not fully capture the inflammatory status of the lung. Identifying appropriate biomarkers during periods of suppressed inflammatory cytokine expression could allow for timely IFN-β administration, which may help restore host defense mechanisms, prevent nosocomial infections, and potentially mitigate the progression of ARDS, thereby serving as a therapeutic intervention.

## Supplementary Information

Supplementary material 1.

Supplementary material 2.

Supplementary material 3.

## Data Availability

Additional details of methods are included in the online supplement. The resulting data are shown as mean or median values, as well as dots for individual data points. The datasets used and/or analyzed during the current study are available from the corresponding author on reasonable request.

## Abbreviations

ARDS: Acute respiratory distress syndrome
LPS: Lipopolysaccharide
IFN: Interferon
CLP: Cecal ligation and puncture
i.t.: Intratracheal
BALF: Bronchoalveolar lavage fluid
IL: Interleukin
TNF: Tumor necrosis factor
KC: Keratinocyte-derived chemokine
MCP-1: Monocyte chemoattractant protein 1
W/D: Wet-to-dry ratio
COVID-19: Coronavirus disease 2019
