# Supplementary material for: Lung immune incompetency after mild peritoneal sepsis and its partial restoration by type 1 interferon: a mouse model study
Source: Intensive Care Med Exp. 2024 Dec 20;12:119. doi: 10.1186/s40635-024-00707-7 (PMC11662124; doi:10.1186/s40635-024-00707-7)
Supplement: Supplementary file 1 — Supplementary material 1. [file 40635_2024_707_MOESM1_ESM.docx]

**SUPPLEMENTAL METHODS**

**Mice**

Male C57/BL6 mice (Jackson Laboratories, Clea Japan, Tokyo, Japan), 8–10 weeks of age and weighing 20–25 g were kept for at least one week on a 12-h light and dark cycle with *ad libitum* feeding. The protocol used for all animal experiments was approved by the University of Tokyo Graduate School of Medicine Institutional Review Board (#Med-P20-106) and was conducted with strict compliance to their ethical guidelines.

**Peritoneal Sepsis**

Under general anesthesia with 2%–4% isoflurane, mice underwent cecal ligation and puncture^1,2^. A midline laparotomy was performed, and the cecum was identified and meticulously exteriorized. Feces were collected from the cecum by gentle squeezing and the cecum was ligated with 4-0 silk thread 1 cm from the tip. The ligated cecum was then punctured with a 23-gauge needle to create two holes. After returning the cecum to the peritoneum, the abdomen was closed in two layers (4-0 silk for the peritoneal membrane and 3-0 silk for the skin), and 1 ml of normal saline containing 25 mg/kg of imipenem-cilastatin (Merck & Co, Kenilworth, NJ, USA) was administered subcutaneously.

The sham surgery group only received laparotomy with cecal exteriorization.

**Interferon-β**

Interferon-β (IFN; R&D Systems, Minneapolis, MN, USA) was diluted to an appropriate concentration in sterile PBS. At 1 h after CLP, the animals received either IFN-β (60 µg/kg, 200 μl) or the same volume of normal saline subcutaneously. Isolated monocytes from mice were incubated on media for 3 h with IFN-β (0.125 µg/ml) before being stimulated with LPS^2,3^.

**Pneumonia** **model**

Four days after CLP, mice were anesthetized under 2% isoflurane. After confirming the optimal anesthetic depth, a 1 cm skin incision was made on the neck to expose the trachea. Then, 50 µl of PBS suspension with 30 µg *Escherichia coli* LPS (O111: B4, Sigma–Aldrich, St. Louis, MO, USA) or sterile PBS alone was instilled intratracheally using a syringe with a 25-gauge needle into CLP-operated or control mice, respectively.

**Septic Acute Lung Injury and Sample Collection**

Four experimental groups were evaluated: sham surgery only, CLP-sepsis only, LPS induced acute lung injury only, and sepsis + LPS induced acute lung injury (ALI). Survival rates were calculated for all groups over the entire 11-day study period after CLP.

The retro-orbital sinus method was used for blood sample collection^4^. Briefly, under anesthesia, a sterile capillary tube was inserted into the medial canthus towards the retro-orbital sinus.

After flushing pulmonary circulation via the right ventricle with ice-cold PBS, the right inferior lobe of lung was excised and transferred into gentleMACS^®^ C Tubes. The lung was enzymatically dissociated using the mouse Lung Dissociation Kit (Miltenyi Biotec, Auburn, CA, USA) using the gentleMACS^®^ Octo Dissociator with a 37°C heater and the gentle rotation program (Miltenyi Biotec). The dissociated lung was applied to a Falcon^®^ 40 µm Cell Strainer to obtain a single-cell suspension. The remaining erythrocytes were lysed with BD PharmLyse™ (BD Biosciences, San Jose, CA, USA). The centrifuged cell pellet was resuspended in flow cytometry staining buffer (R&D SYSTEMS, Minneapolis, MN, USA) and EasySep™ Buffer (STEMCELL, Vancouver, BC, Canada) for flow cytometry and immunomagnetic cell isolation after manual cell counting.

Histological evaluation of the lungs was conducted on harvested lungs before and 4-day after sepsis, and 1-day after acute lung injury induced by intratracheal LPS.

**Bronchoalveolar Lavage Fluid (BALF) Collection**

For bronchoalveolar lavage, a 22-gauge catheter was intubated into the trachea and 0.5 ml of PBS containing 1 mM EDTA was injected and withdrawn three times, and this process was repeated three times^5^. The recovered BALF was centrifuged to pellet cells. After passing through a 0.22-μm filter, the protein concentration in the supernatant was measured using a Protein Assay BCA Kit (FUJIFILM, Osaka, Japan) and cytokine analysis. The pellet was resuspended in 500 μl of PBS and the total cell numbers were counted manually.

**Flow Cytometry**

Fluorophore-conjugated antibodies comprised (Supplemental Table E1): anti-CD45 (Clone BM8, Biolegend, San Diego, CA), anti-CD11b (Clone M1/70, Biolegend), anti-CD11c (Clone N418, Biolegend), anti-SiglecF (Clone E 50-2440, BD Biosciences, Franklin Lakes, NJ), anti-Ly6G (Clone 1A8, Biolegend), anti-Ly6C (Clone HK1.4, Biolegend), anti-CD115 (Clone AFS98, Biolegend), anti-MHCII (Clone REA813, Miltenyi Biotec), anti-CD3 (Clone 17A2, Biolegend), and anti-CCR2 (Clone SA203G11, Biolegend). Target cell populations were identified according to a gating strategy for the mouse lung. Surface markers were quantified on a flow cytometer CytoFLEX (Beckman Coulter, Brea, CA, USA) and analyzed with CytExpert 2.4 software. Exact cell numbers were determined by the total number and percentage of each cell population.

**Cytokine Analysis**

Cytokines (IL-6, IL-10, TNF-α, KC, and MCP-1) in serum, BALF, and the lung interstitium monocyte media supernatant were evaluated by flow cytometer with the BD™ Cytometric Bead Array kit (Becton, Dickinson and Company, Franklin Lakes, NJ, USA) according to the manufacturer’s instructions.

**mRNA Expression Assay**

The left lung from each mouse was homogenized in lysis buffer using GentleMACS® M Tubes. Total RNA from lung tissue or isolated monocytes was extracted using the GenElute Mammalian Total RNA Miniprep Kit (Sigma–Aldrich) according to the manufacturer’s instructions. cDNA synthesis was performed with the Transcriptor Strand cDNA Synthesis Kit (Roche Diagnostics, Mannheim, Germany). The expression levels of *ccl2*, *cxcl1*, *il6*, *tnf*, and *il10* were quantified by a TaqMan™ Gene expression assay (Applied Biosystems, Framingham, MA, USA) using the StepOne™ Real-Time PCR System (Applied Biosystems). TaqMan™ probes for each cytokine are summarized in Supplemental Table E2. Expression values for each gene were normalized to that of the *gapdh* gene, and results were expressed as fold changes relative to unstimulated controls for each condition.

**RNA-sequencing**

Total RNA was extracted from the left lungs using the phenol-chloroform method. RNA quality and integrity were assessed with a Bioanalyzer, ensuring an RNA integrity number greater than 8. mRNA was enriched using the NEBNext® Poly(A) mRNA Magnetic Isolation Module (New England Biolabs, Ipswich, MA, USA), according to the manufacturer’s instructions.

Strand-specific RNA libraries were prepared with the NEBNext® Ultra™ II Directional RNA Library Prep Kit (New England Biolabs). The mRNA was reverse-transcribed into first-strand cDNA, followed by second-strand synthesis using dUTP to retain strand specificity. After end repair, phosphorylation, and 3'-dA tailing, sequencing adapters containing dUTP were ligated. The dUTP-labeled second strand was selectively degraded, leaving single-stranded cDNA for high-throughput sequencing.

The cDNA libraries were sequenced on an Illumina NovaSeq 6000 platform to generate paired-end reads. Raw sequencing data underwent quality control using FastQC (Version 0.11.7), and low-quality bases and adapter sequences were trimmed with Trimmomatic (Version 0.38). High-quality reads were aligned to the reference genome using HISAT2 (Version 2.1.0) and gene expression levels were quantified with featureCounts (Version 1.6.3).

**Lung Wet-to-dry Weight (W/D) Ratios**

Lung wet/dry (W/D) weight ratios were measured as described previously^6^. The left lobes of lung tissues were dissected and weighed immediately. The lung tissues were then desiccated at 60°C for 48 h and weighed.

**Histological Examination and Scoring**

The lung tissues were fixed in 4% paraformaldehyde and paraffin embedded. The tissue sections (3 µm) were stained with hematoxylin and eosin, examined under a light microscope (Keyence, Tokyo, Japan), and photo images were recorded. The lung injury score (LIS) was determined according to the lung injury scoring system of the American Thoracic Society^7^. Twenty random high-power ﬁelds (400× total magniﬁcation) were independently scored for each condition.

**Isolation of Tissue Monocytes in the** **Lung and Evaluation of their Function**

A single-cell suspension was prepared at a concentration of 1×10^8^ cells/ml. Highly purified monocytes were isolated using an immunomagnetic negative selection kit (ST-19861, *EasySep*™, STEMCELL). Cells other than monocytes were supplemented with antibodies targeting their respective surface antigens and captured on magnetic beads. Isolated monocytes were seeded at 5 x 10^5^/500 µl in Roswell Park Memorial Institute medium and incubated for 2 h after quantifying the surface antigen by flow cytometry. After washing, cells were stimulated with 10 ng/ml of *E. coli* LPS for 3 h. Cytokine secretion and monocyte mRNA expression in response to LPS were evaluated with the BD™ Cytometric Bead Array assay and real-time PCR separately.

**Experimental Units**

A cage of animals

**Sample Size Calculation**

Based on the results of our previous study^3^, assuming a survival probability per day of 0.85 for the severe CLP group and 0.9999 for the control group, with a 7-day observation period, an alpha value of 0.05 and 1-β = 0.8, the sample size was calculated by the Lakatos' method^8^ to be 8 for each group.

**Murine Sepsis Score**

Murine sepsis scores were monitored every 12 h according to the table including seven parameters consisting of spontaneous activity, response to touch and auditory stimuli, posture, respiration rate and quality (labored breathing or gasping), and appearance (i.e., degree of piloerection)^9^. The total score was calculated by assigning a score of 0-4 to each item, with a minimum of 0 and a maximum of 28.

**Humane Endpoints after CLP**

Animals were euthanized if the total murine sepsis score was 21 or more^9^, or if the respiratory rate or respiratory quality score was 3 or more. The above exclusion criteria were established *a priori*.

**Randomization**

A cage of animals (3 mice / cage) was randomly allocated to either the sham surgery, CLP, or CLP + IFNβ group. Cages were selected by a blinded researcher. No random-numbers table was used.

**Blinding**

After allocation, one researcher conducted the surgery indicated on each cage, up until sample collection. Sample processing was conducted by another researcher blinded to the group allocation. Data were analyzed by the two researchers, along with an additional researcher. The number of mice used in a single experimental series was kept to no more than 30 to minimize confounding factors due to the significant time lag between therapeutic measures.

**SUPPLEMENTAL FIGURE LEGENDS**

**Supplemental figures.**

**Supplemental Figure** **E1**. Reproduction of sepsis-related acute lung injury in mice. (A) Kaplan–Meier survival analysis of mice induced with sepsis by CLP (Hit 1), then pneumonia by i.t. instillation of LPS (30 µg in 50 µl PBS; Hit 2); CLP group mice (n = 80) underwent CLP and i.t. instillation of saline; Lung LPS group mice (n = 40) underwent sham laparotomy instead of CLP, followed by LPS administration; CLP 🡪 Lung LPS group mice (n = 50) underwent CLP followed by LPS administration. All mice received broad-spectrum antibiotics (ATB) by subcutaneous injection within 1 h of CLP or sham surgery. All three groups showed over 90% survival, with no statistically significant differences. Data represent the combined results of three independent experiments, each with similar results. (B) Postoperative change in body weight (BW) in the three groups, showing a continuous decline until day 3 in the CLP group. Most of the mice started to regain their body weight thereafter. Each black line tracks the weight change of a single mouse from Day 0 (Pre-CLP) to Day 6, while the red line indicates the average weight across all mice at each time point. (C) Clinical severity scores, with the CLP group showing an initial high score dropping to almost zero (no clinical severity) on day 4.

**Supplemental Figure E2**. Systemic cytokine profile before (Pre-CLP) and after CLP-induced sepsis. Serial serum samples were harvested before CLP and on Days 2, 3, 4, and 5 after CLP. Cytokines / chemokine levels (TNF-α, IL-6, MCP-1, KC and IL-10) were evaluated by flow cytometry with a BD™ Cytometric Bead Array kit (Becton, Dickinson and Company, Franklin Lakes, NJ, USA) according to the manufacturer’s instructions. Data are expressed as the mean ± SD, accompanied by individual data points. Exact *P* values are shown for each comparison.

**Supplemental Figure E3**. Flow-based detection of immune cells from a whole lung cell suspension enzymatically digested from the excised right inferior lobe of the lung.

E2A; (a) Scattergram of the whole lung cells. (b) Cell differentiation using CD11b and Siglec-F. (c) CD11b+ and Sglec-F+ cells for alveolar macrophages. (d) CD11b- and CD3+ cells for lymphocytes. (e) CD11b+ and Ly6G- cells for monocytes and CD11b+ and Ly6G+ cells for neutrophils.

E2B; Time course cytogram of the whole lung cells stained with anti-CD11b and anti-Ly6G antibodies. (a) Before CLP, (b) Day 2 after CLP, (c) Day 3 after CLP, (d) Day 4 after CLP. Each panel consists of 10,000 cells. Note that neutrophils (CD11b+, Ly6G+) and monocytes (CD11b+, Ly6G-) increased after CLP-induced sepsis.

**Supplemental Figure E4**. Myeloid cell populations in the bronchoalveolar lavage fluid (BALF). Myeloid cells among the bronchoalveolar lavage cells were enumerated by flow cytometry. (A) Neutrophils; neutrophils were almost absent without i.t. LPS in both the CLP and noCLP groups. However, the majority of the myeloid cells were migrated neutrophils after i.t. LPS. (B) Alveolar macrophages; without i.t. LPS, the majority (>90%) of the lavage cells were alveolar macrophages in both the CLP and noCLP groups. (C) Monocytes; monocyte numbers in the lavage were similar in both the CLP and noCLP groups without i.t. LPS. However, a significantly larger number of monocytes migrated into the alveoli after i.t. LPS in the CLP group. Data are expressed as the median ± [25th, 75th percentile], accompanied by individual data points. Exact *P* values are shown for each comparison.

**Supplemental Figure E5**. Flow-based detection of monocytes among the whole lung cells. Enzymatically isolated monocytes from the whole lung cells were detected as CD115+. Monocyte populations were further characterized by surface antigen expression, i.e., Ly6C and CCR2. On Day 4 after CLP, the Ly6C- population increased compared with noCLP mice. Note that Ly6C^hi^ monocytes were also CCR2+.

**Supplemental Figure E6**. Systemic cytokine profile before and 1-day after i.t. LPS in the CLP and noCLP groups. Serum samples were harvested on the indicated days and cytokines / chemokine levels (TNF-α, IL-6, MCP-1, KC, and IL-10) were evaluated by flow cytometry with the BD™ Cytometric Bead Array kit (Becton, Dickinson and Company) according to the manufacturer’s instructions. The systemic cytokine response after i.t. LPS was blunted in the CLP group compared with the noCLP group except for TNF-α. The IL-10 level was below the detection limit in all samples, so a bar graph is not shown. Data are expressed as the median ± [25th, 75th percentile], accompanied by individual data points. Exact *P* values are shown for each comparison.

**Supplemental Figure E7**. Effect of sepsis on lung immune-suppressive mRNA expression. All evaluated mRNAs exhibited a significant reduction 4 days after CLP compared with the control (Pre-CLP), except for SOCS3, which showed a significant increase. Data are expressed as the median ± [25th, 75th percentile], accompanied by individual data points. Exact *P* values are shown for each comparison.

**Supplemental Figure E8**. Schematic illustrating the seemingly contradictory phenomena found in this study. On the one hand, sepsis causes disruption of vascular endothelial and alveolar epithelial integrity, leading to increased numbers of leukocytes (monocytes, neutrophils) migrating into the interstitium and alveoli after the 2nd hit (i.t. LPS), and protein-rich edematous fluid accumulating in the alveoli (histological impression = hyperinflammation). On the other hand, after sepsis, Ly6C^lo^ monocytes accumulate in the pulmonary interstitium over time, and inflammatory cytokine expression in the lung tissue is suppressed. 2nd hit causes influx of Ly6C^hi^ monocytes into the alveoli, but they are unable to express effective inflammatory cytokines in the alveoli, resulting in a state of immune paralysis (impression from immunologically regulator function = hypoinflammation). This state of immunological paralysis may increase the damage of the 2nd hit due to the impaired host-defense capacity. ROS; reactive oxygen species.

**References**

1. Rittirsch D, Huber-Lang MS, Flierl MA, Ward PA. Immunodesign of experimental sepsis by cecal ligation and puncture. Nat Protoc 2009;4:31-6.

2. Hiruma T, Tsuyuzaki H, Uchida K, et al. IFN-β improves sepsis-related alveolar macrophage dysfunction and postseptic acute respiratory distress syndrome-related mortality. Am J Respir Cell Mol Biol 2018;59:45–55.

3. Kusakabe Y, Uchida K, Yamamura Y, et al. Early-phase innate immune suppression in murine severe sepsis is restored with systemic interferon-β. Anesthesiology 2018;129:131–42.

4. Teilmann AC, Madsen AN, Holst B, Hau J, et al. Physiological and pathological impact of blood sampling by retro-bulbar sinus puncture and facial vein phlebotomy in laboratory mice. PLoS ONE 2014;9:e113225.

5. Hoecke L van, Job ER, Saelens X, Roose K. Bronchoalveolar lavage of murine lungs to analyze inflammatory cell infiltration. J Vis Exp 2017;123:55398.

6. Folz RJ, Abushamaa AM, Suliman HB. Extracellular superoxide dismutase in the airways of transgenic mice reduces inflammation and attenuates lung toxicity following hyperoxia. J Clin Invest 1999;103:1055-66.

7. Matute-Bello G, Downey G, Moore BB,et al. An official american thoracic society workshop report: Features and measurements of experimental acute lung injury in animals. Am J Respir Cell Mol Biol 2011;44:725-38.

8. Lakatos E. Sample sizes based on the log-rank statistic in complex clinical trials. Biometrics 1988;44:229-41.

9. Shrum B, Anantha RV, Xu SX, et al. A robust scoring system to evaluate sepsis severity in an animal model. BMC Res Notes 2014;7:1-11.
