# Supplementary figures and images for: Lung immune incompetency after mild peritoneal sepsis and its partial restoration by type 1 interferon: a mouse model study
Source: Intensive Care Med Exp. 2024 Dec 20;12:119. doi: 10.1186/s40635-024-00707-7 (PMC11662124; doi:10.1186/s40635-024-00707-7)

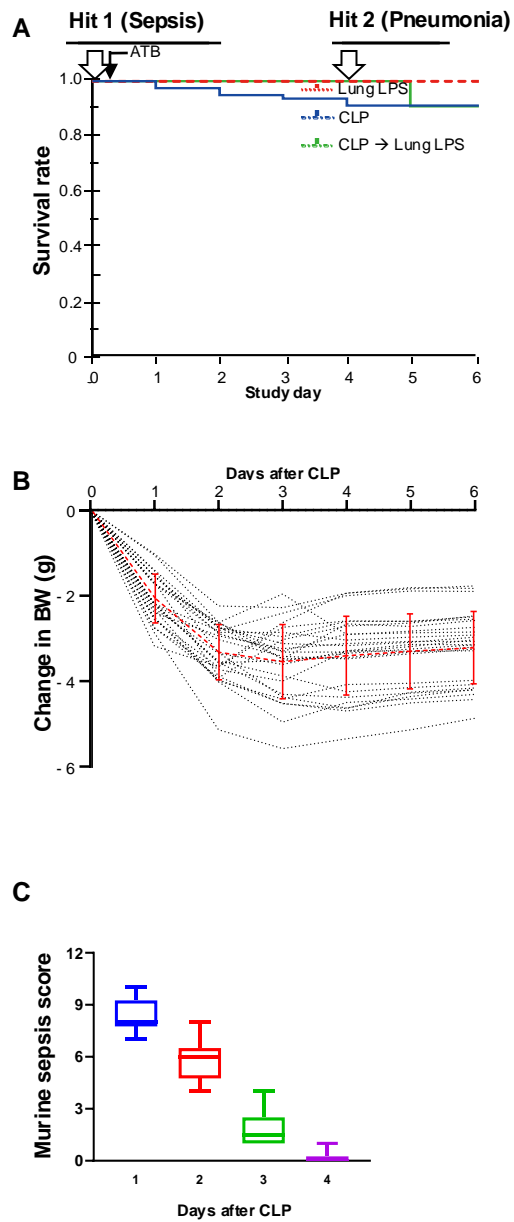

**Fig. E1**

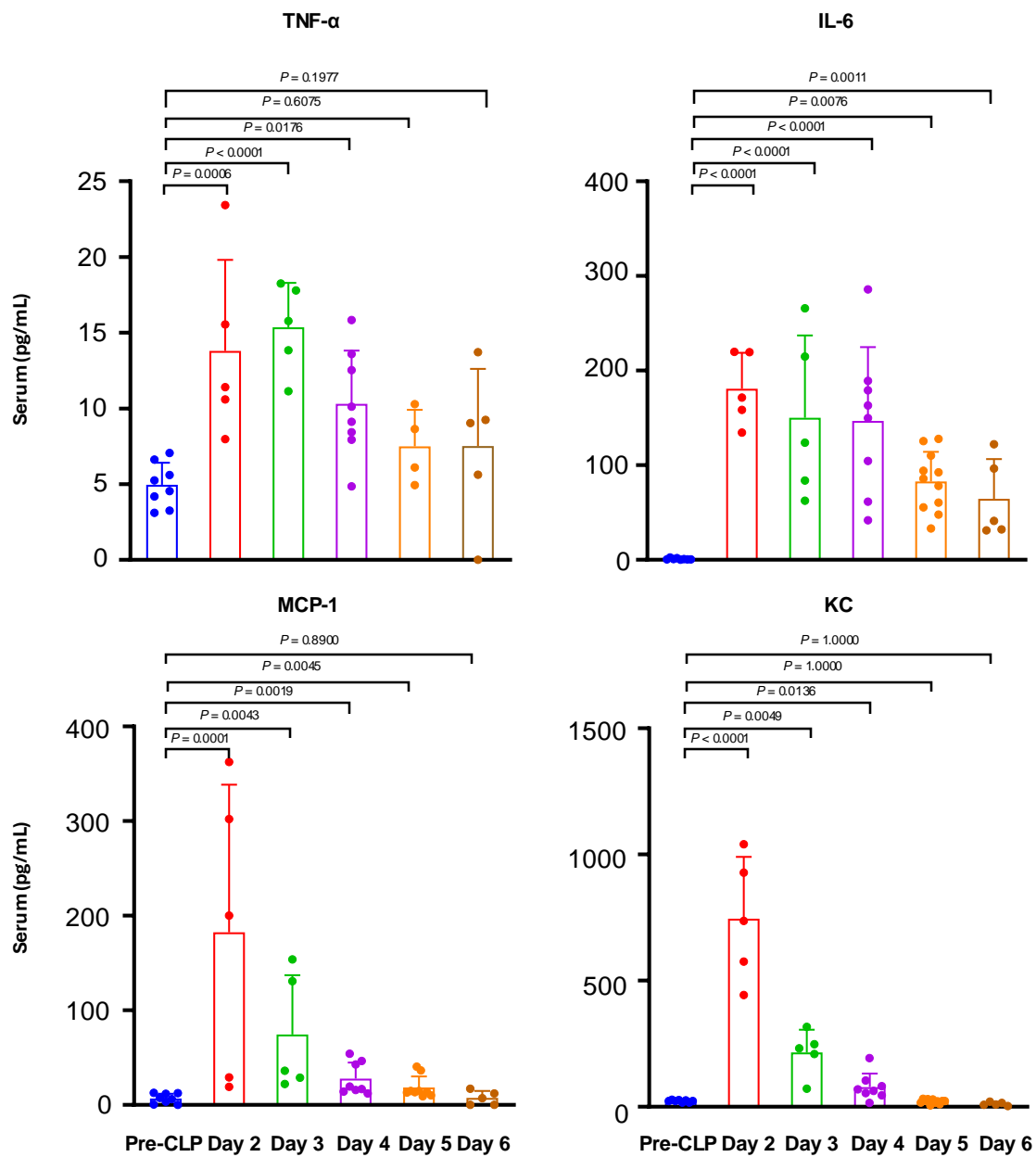

**Fig. E2**

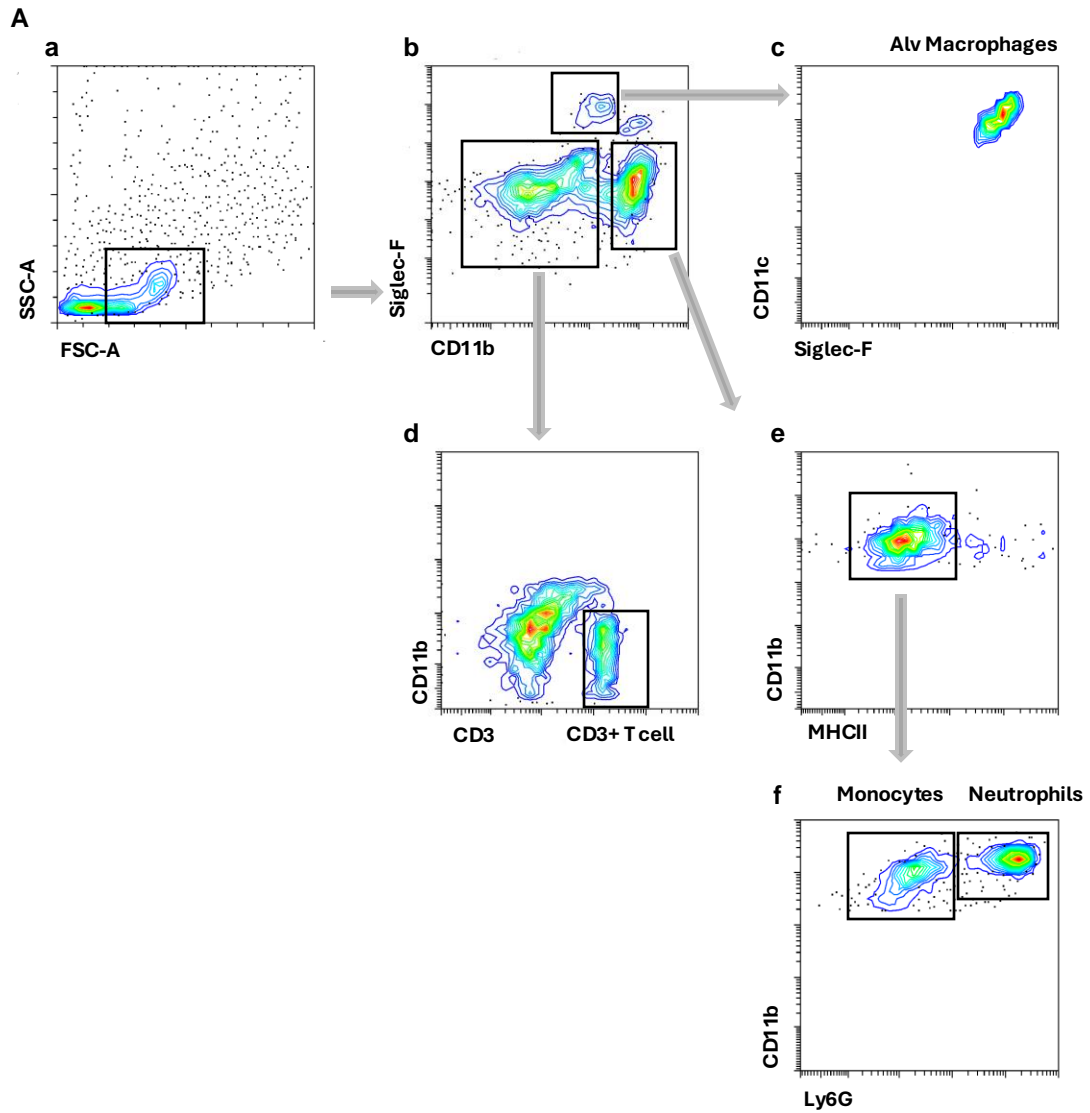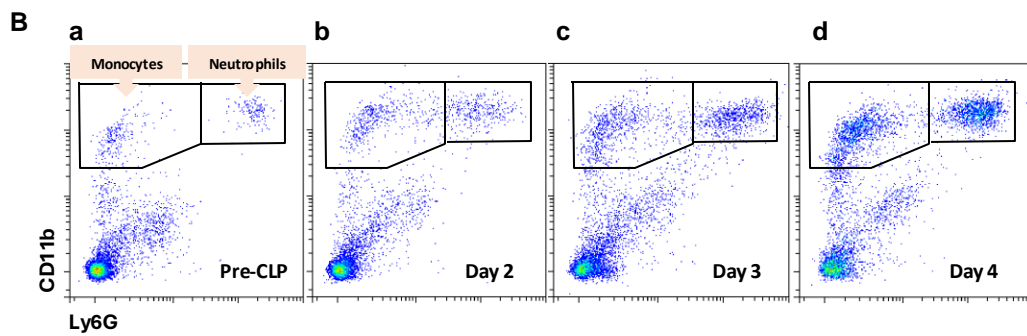

**Fig. E3**

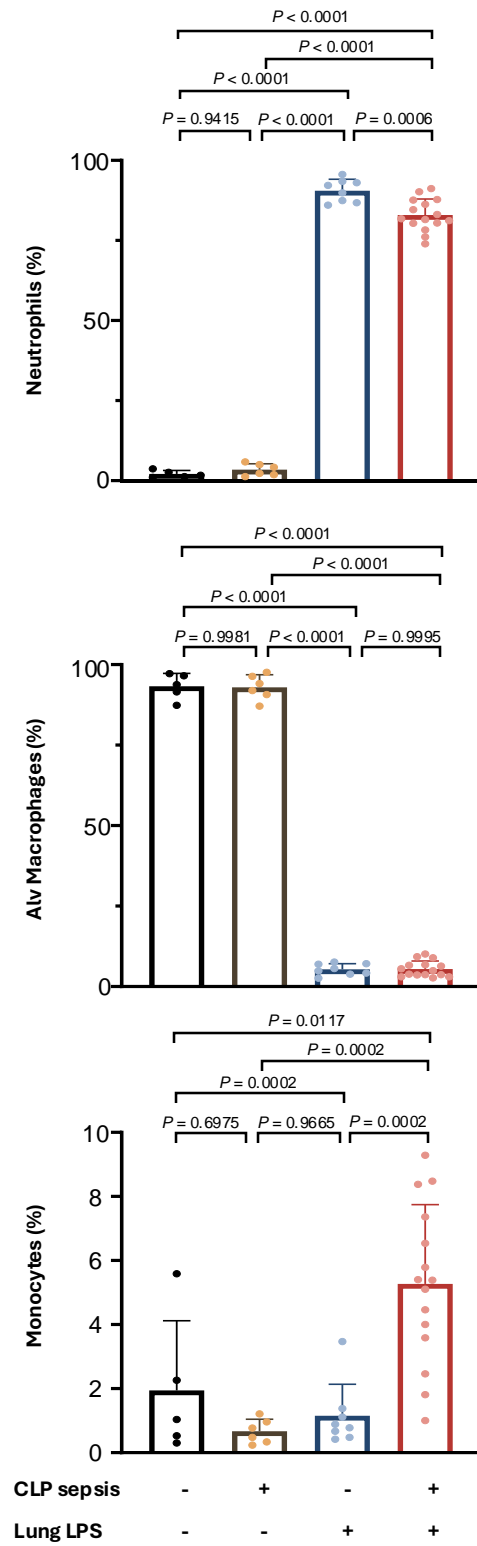

**Fig. E4**

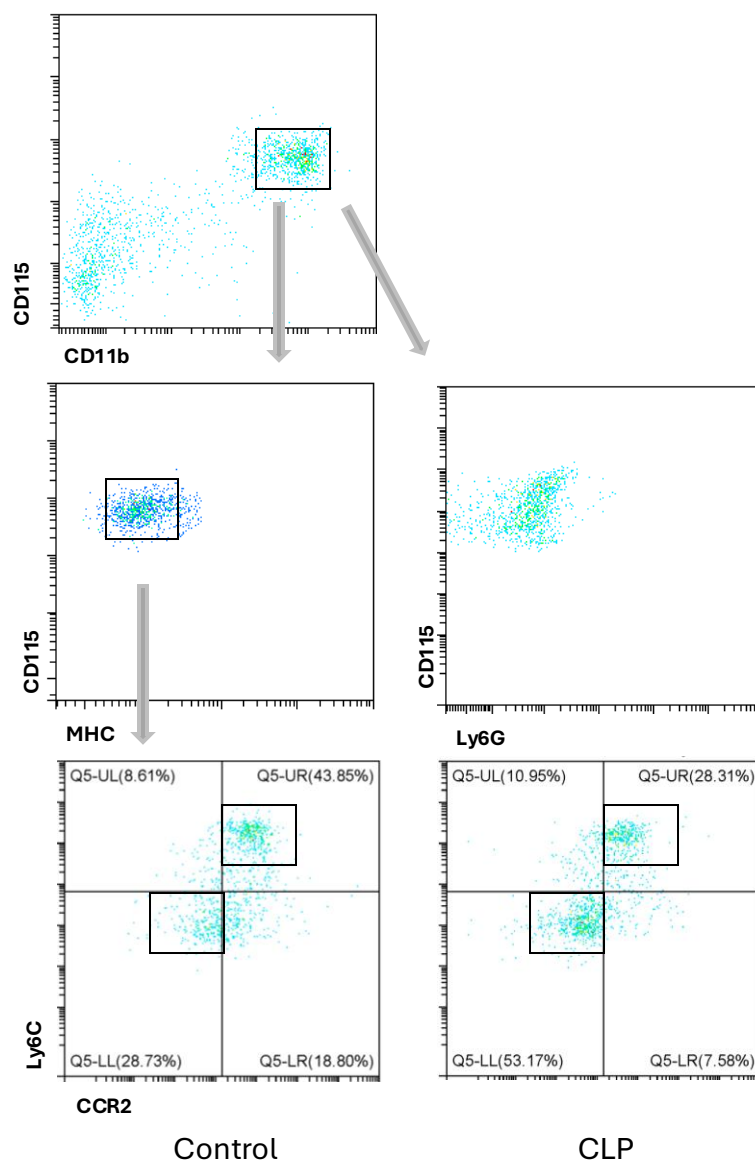

**Fig. E5**

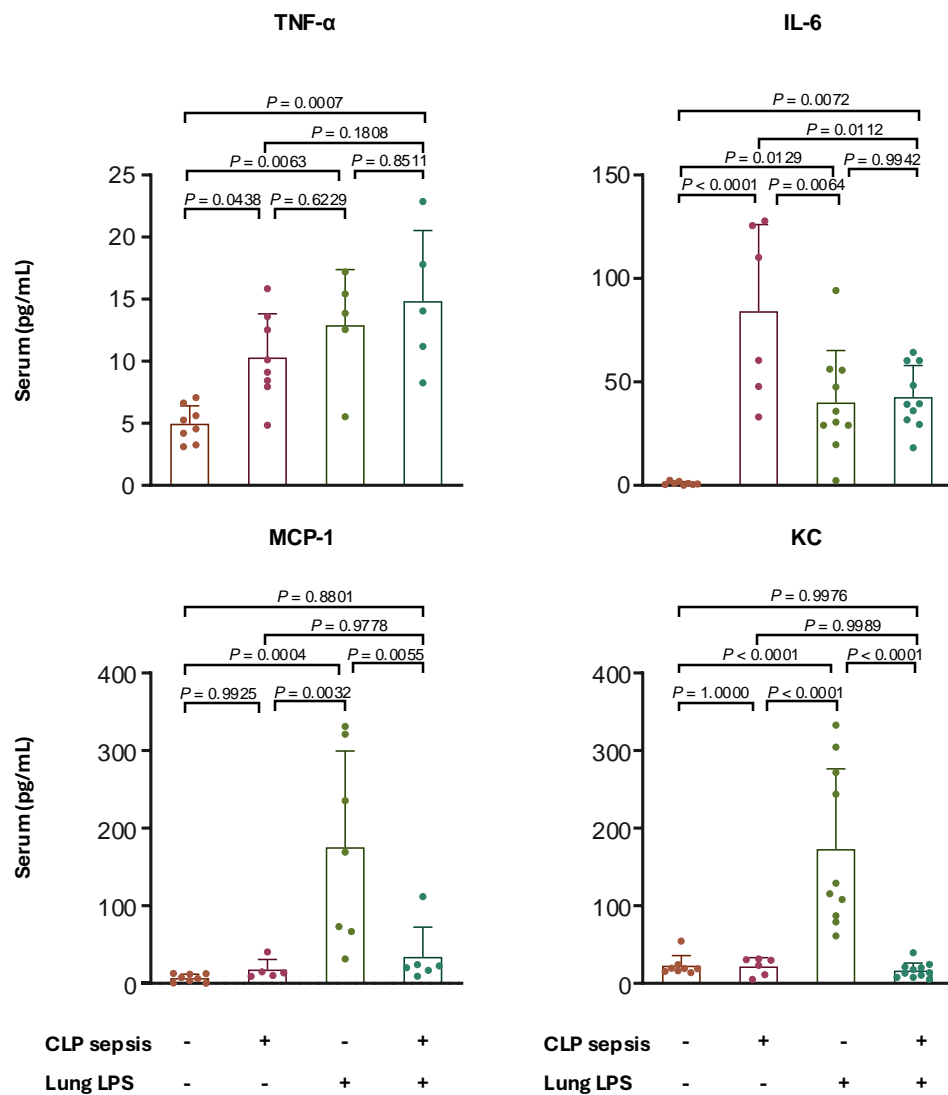

**Fig. E6**

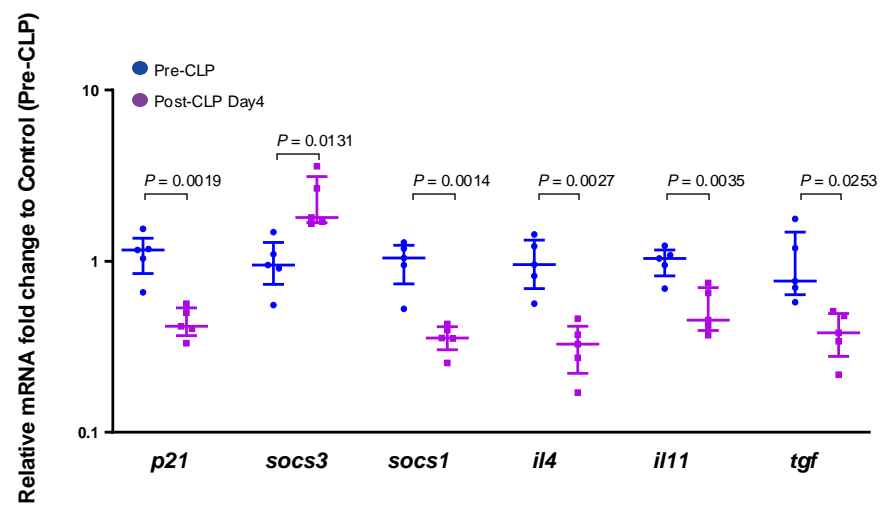

Fig. E7

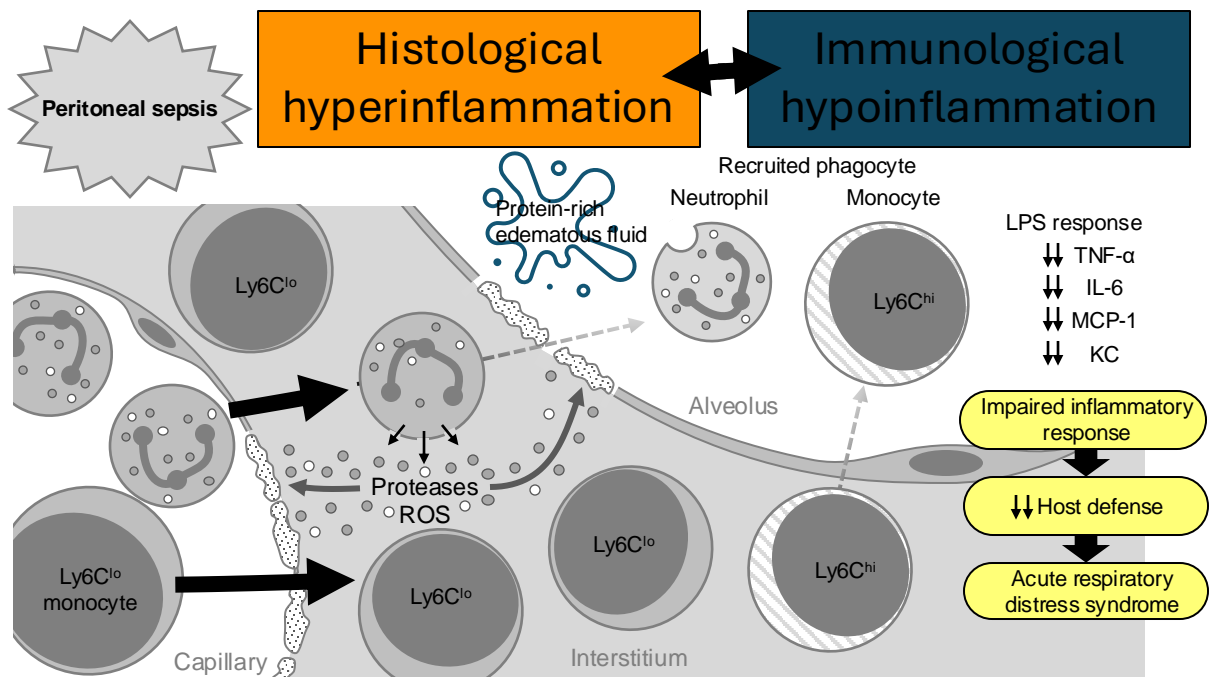

**Fig. E8**

Supplement: Supplementary file 2 — Supplementary material 2. [file 40635_2024_707_MOESM2_ESM.pdf]
