# Supplementary material for: Lung immune incompetency after mild peritoneal sepsis and its partial restoration by type 1 interferon: a mouse model study
Source: Intensive Care Med Exp. 2024 Dec 20;12:119. doi: 10.1186/s40635-024-00707-7 (PMC11662124; doi:10.1186/s40635-024-00707-7)
Supplement: Supplementary file 3 — Supplementary material 3. [file 40635_2024_707_MOESM3_ESM.pdf]

Target Antigen	Clone	Manufacturer
CD11b	M1/70	Biolegend
Ly6G	1A8	Biolegend
Ly6C	HK1.4	Biolegend
CD3	17A2	Biolegend
SiglecF	E50-2440	BD Pharmingen
MHC2	REA813	Miltenyi
CD45	BM8	Biolegend
CD11c	N418	Biolegend
CD86	GL-1	Biolegend
CD115	AFS98	Biolegend
CCR2	SA203G11	Biolegend

Table E1

Fluorochrome-conjugated antibodies used to detect cell surface antigen for Flow cytometry

Target Gene	TaqMan® Probe
gapdh	Mm99999915_g1 Gapdh
tnf	Mm00443258_m1 Tnf
il6	Mm00446190_m1 Il6
cxcl1	Mm04207460_m1 Cxcl1
ccl2	Mm00441242_m1 Ccl2
il10	Mm00439614_m1 Il10

Table E2

TaqMan™ probe used for mRNA quantification
